# Functional relationship between peripheral thermosensation and behavioral thermoregulation

**DOI:** 10.3389/fncir.2024.1435757

**Published:** 2024-07-09

**Authors:** Takuto Suito, Makoto Tominaga

**Affiliations:** ^1^Division of Cell Signaling, National Institute for Physiological Sciences, National Institutes of Natural Sciences, Okazaki, Japan; ^2^Exploratory Research Center on Life and Living Systems, National Institutes of Natural Sciences, Okazaki, Japan; ^3^Nagoya Advanced Research and Development Center, Nagoya City University, Nagoya, Japan

**Keywords:** TRP channel, thermosensation, behavioral thermoregulation, temperature preference, *Drosophila melanogaster*

## Abstract

Thermoregulation is a fundamental mechanism for maintaining homeostasis in living organisms because temperature affects essentially all biochemical and physiological processes. Effector responses to internal and external temperature cues are critical for achieving effective thermoregulation by controlling heat production and dissipation. Thermoregulation can be classified as physiological, which is observed primarily in higher organisms (homeotherms), and behavioral, which manifests as crucial physiological functions that are conserved across many species. Neuronal pathways for physiological thermoregulation are well-characterized, but those associated with behavioral regulation remain unclear. Thermoreceptors, including Transient Receptor Potential (TRP) channels, play pivotal roles in thermoregulation. Mammals have 11 thermosensitive TRP channels, the functions for which have been elucidated through behavioral studies using knockout mice. Behavioral thermoregulation is also observed in ectotherms such as the fruit fly, *Drosophila melanogaster*. Studies of *Drosophila* thermoregulation helped elucidate significant roles for thermoreceptors as well as regulatory actions of membrane lipids in modulating the activity of both thermosensitive TRP channels and thermoregulation. This review provides an overview of thermosensitive TRP channel functions in behavioral thermoregulation based on results of studies involving mice or *Drosophila melanogaster*.

## 1 Introduction

Sensory function is fundamental for organisms to perceive external environmental cues that enable maintenance of homeostasis through appropriate responses to changes in the environment. Temperature is a crucial environmental factor that affects a range of various physiological processes through impacts on enzyme activities and biochemical reactions. Additionally, temperature functions as a signaling factor in the development and reproduction of a range of organisms (Hammel, 1968).

For animals, a key response to environmental temperature changes is thermoregulation, which can be categorized as physiological and behavioral. Physiological thermoregulation is observed mainly in homeotherms, and includes shivering thermogenesis in skeletal muscles, non-shivering thermogenesis in brown adipose tissue, and vasodilation/vasoconstriction. Behavioral thermoregulation is a crucial, conserved physiological function for both homeotherms and ectotherms that includes seeking comfortable temperature zones, avoiding extreme temperatures, and posture changes (Tan and Knight, 2018).

At the forefront of these thermoregulatory activities is thermosensation in the peripheral nervous system, which involves temperature receptors such as Transient Receptor Potential (TRP) channels that are expressed on the cell membrane of sensory neurons. For physiological thermoregulation, peripheral thermosensation regulates heat production through shivering and non-shivering thermogenesis. These pathways are well-characterized, particnularly in mice (Morrison and Nakamura, 2011). However, the neuronal pathways that are involved in behavioral thermoregulation remain less well understood. This review outlines the role of temperature receptors, particularly TRP channels, in behavioral thermoregulation.

## 2 Thermosensitive TRP channels

**Figure 1:**
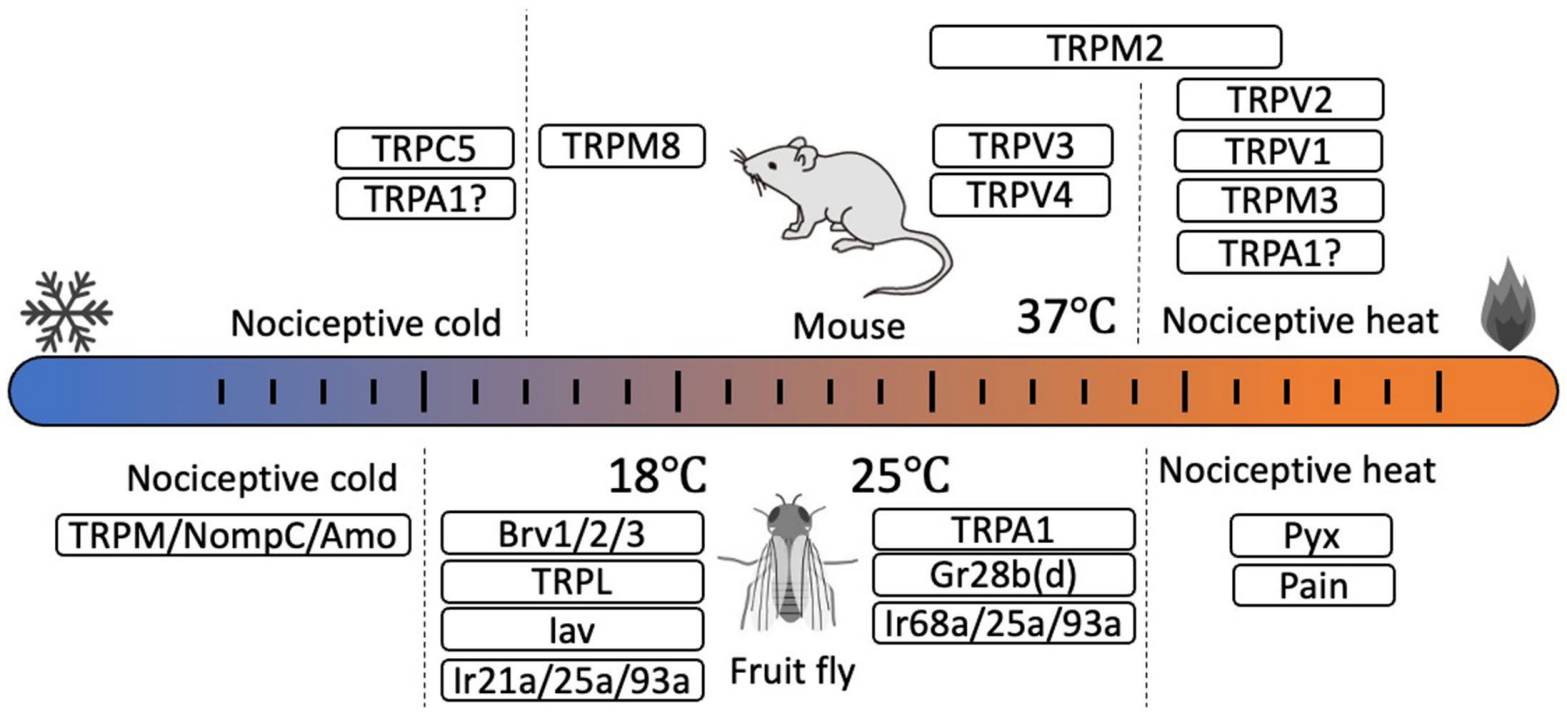
Thermosensors for somatic thermosensation and behavioral thermoregulation identified in mouse (*Mus musculus*, upper) and fruit fly (*Drosophila melanogaster*, lower). In mice, several thermosensitive TRPs are identified as thermosensors. TRPV1, TRPV2, and TRPM3 are involved in nociceptive heat sensation while TRPM8 is a sensor of innocuous cold. TRPC5 in odontoblasts is involved in nociceptive cold detection. TRPV3 and TRPV4 are activated at near body temperature. The thermosensory role of TRPA1 remains controversial. In *Drosophila*, involvement of several TRPs, ionotropic receptors (Irs), and gustatory receptor (Gr) in thermosensation and temperature preference have been identified. However, the temperature sensitivity of cold temperature receptors has not yet been determined.

TRP channels are non-selective cation channels that have six transmembrane domains and function as tetrameric polypeptides. TRP channels are categorized into seven subfamilies (six in mammals) based on the differences in amino acid sequence, especially in the intracellular N- and C-terminal domains. Various physical and chemical signals activate TRP channels as part of numerous physiological pathways (Venkatachalam and Montell, 2007). In mammals, 11 TRP channels are reported to be thermosensitive, called thermo-TRPs (Xiao and Xu, 2021; Kashio and Tominaga, 2022) (Figure 1). TRPV1 was the first temperature receptor to be identified, and is activated by heat stimulus above 43°C. Thus, TRPV1 functions as a receptor for noxious heat stimuli in sensory neurons (Caterina et al., 1997). TRPV2 (Caterina et al., 1999) and TRPM3 (Vriens et al., 2011) also act as receptors for noxious heat stimuli. On the other hand, TRPM8 functions in cold sensation, with an activation threshold temperature below 27°C (Bautista et al., 2007; Dhaka et al., 2007). TRPC5 is activated by low-temperature stimuli below 14°C, and TRPC5/TRPA1 double knockout mice show a lack of pain response to cold stimuli sensed by trigeminal neurons (Bernal et al., 2021). TRPA1 was initially sensing noxious cold stimuli in mice (Story et al., 2003), but a recent analysis using TRPV1/TRPM3/TRPA1 triple knockout mice indicated that TRPA1 is involved in sensing noxious heat stimuli as evidenced by the complete loss of responses to noxious heat stimuli by these mice, even though responses by mice with single or double TRP channel knockout were not completely abolished (Vandewauw et al., 2018). Studies on human TRPA1 also reported channel activation at both high and low temperatures, even in the absence of the N-terminal domain (Moparthi et al., 2022). In contrast to mammalian TRPA1, TRPA1s in birds, reptiles, amphibians, and insects are reported to exhibit clear high temperature sensitivity (Saito and Tominaga, 2015). Although the thermosensitivity of mammalian TRPA1 remains controversial, the potential heat sensitivity of mammalian TRPA1 could reveal novel functional roles for this channel in temperature-dependent physiological events.

At near body temperature, TRPM2, TRPV3, and TRPV4 function as temperature sensors. TRPM2 is widely expressed in various tissues, but in sensory neurons TRPM2 functions independently of TRPV1 and TRPM3 in warm-temperature perception (Tan and McNaughton, 2016). The 47°C temperature threshold of naïve TRPM2 can be reduced by various intracellular signals such as ADP ribose and reactive oxygen species, thus enabling TRPM2 activation at near body temperature (Kashio et al., 2022). Additionally, TRPM2 is reported to suppress excessive temperature elevations at the preoptic area during infection-induced fever (Song et al., 2016). TRPV4 is also expressed in various tissues, including sensory neurons, and is activated not only by temperature (Lee H. et al., 2005), but also by various physical stimuli to regulate various cellular functions. Although TRPV3 is not expressed in sensory neurons (Peier et al., 2002), in skin keratinocytes activation of TRPV3 by warm temperature induces calcium influx that initiates ATP secretion that is involved in temperature sensation associated with activation of ATP receptors in sensory neurons ((Mandadi et al., 2009) see also below).

Thermo-TRPs which are involved in thermosensation and thermoperception are mainly expressed in primary sensory neurons such as dorsal root ganglia and trigeminal ganglion in mammals. These neurons project to the cortex through the spinal cord grey matter. Thermosensation mediated by thermosensitive TRPs plays important roles not only in sensory perception through the cerebral cortex but also in temperature-dependent responses in the local organs. For example, we have reported changes in temperature-dependent activity of microglia (Feng et al., 2020; Nishimoto et al., 2021) and responses in secretory organs (Derouiche et al., 2018; Kashio et al., 2024), which shows the cell autonomous temperature responses mediated by thermosensitive TRPs expressed in non-sensory neurons.

## 3 Peripheral thermosensors and behavioral thermoregulation

Many TRP receptors have been characterized as thermoreceptors in behavioral experiments such as a two temperature choice assay and a linear temperature gradient assay with knockout mice. Comprehensive preference temperature analyses of TRPA1, TRPM2, TRPM8, TRPV1, TRPV3, and TRPV4 knockout mice were carried out using a circular continuous thermal gradient device termed Thermal Gradient Ring (TGR) (Ujisawa et al., 2022, 2024). The TGR minimizes the influence of preferential behavior to seek corners that is observed using rectangular devices. For a TGR having a temperature range between 11.5°C and 43.6°C, wild-type mice increased their time spent in an area having a narrow temperature range near 35°C, and spent substantially less time in areas where the temperatures were below 20°C or above 40°C. Mice lacking the cold receptor TRPM8 spent increased amounts of time in areas having a temperatures below 35°C, with intrusion observed into areas having temperatures below 20°C. Mice lacking TRPM2 spent time in areas having a wider temperature range (32°C to 37°C) compared to wild-type mice. Interestingly, mice lacking either TRPV3 or TRPV4, which is estimated to be involved in temperature sensing at near body temperature, showed increased time spent in areas at temperatures below 32°C, a value that does not apparently correlate with the putative activation temperatures of either of these channels. Mice lacking the heat receptor TRPV1 showed increased time spent at temperatures above 45°C in a TGR ranging from 14.6°C to 54.9°C that includes the noxious heat range. Meanwhile, mice lacking TRPA1, the cold sensitivity of which is controversial, did not spend increased amounts of time in colder temperature ranges compared to wild-type mice. These mice did, however, spend increased time at temperatures above 45°C compared to wild-type mice in a TGR ranging from 14.6°C to 54.9°C, supporting a role for TRPA1 in heat sensation.

For the nociceptive temperature response, thermo-TRPs such as TRPV1 and TRPA1 are expressed in C-fibers and/or Aδ-fibers of primary sensory neurons, which orchestrate rapid neural signal transmission. In contrast, perception of temperatures near physiological levels does not necessarily require fast transmission speeds. Recent experiments using TGR emphasized roles for TRPV3 expressed in non-neural tissue in thermosensation and behavioral thermoregulation (Lei et al., 2023). TRPV3 is reported to be expressed in keratinocytes rather than sensory neurons and its cell surface expression is negatively regulated by TMEM79, which is highly expressed in keratinocytes. Interestingly, as mentioned above, TRPV3 knockout mice tend to choose lower temperatures, whereas TMEM79 knockout mice that have elevated skin TRPV3 expression levels exhibit a preference for higher temperature ranges compared to wild-type mice (Lei et al., 2023). These results suggest that temperature input from keratinocytes also contributes to environmental temperature sensation and behavioral thermoregulation. Together, the results of temperature preference tests involving mice having knockout of thermosensitive TRPs indicate the importance of temperature receptor function in behavioral thermoregulation.

## 4 Thermosensation in a *Drosophila* model

Behavioral thermoregulation plays much more significant roles for ectotherms than homeotherms. As body temperature in ectotherms depends on the environmental temperature, these organisms must seek favorable temperature zones to regulate body temperature (Angilletta et al., 2002). This temperature preference or thermotaxis has been used as an index to study thermosensory functions in model organisms like *Drosophila melanogaster* (fruit fly) and *Caenorhabditis elegans* (nematodes) (Angilletta et al., 2002). In insects, and particularly in *Drosophila,* various genes related to temperature sensation, including TRP channels, have been identified (Montell, 2021; Liu and Zhang, 2022) (Figure 1). Moreover, mechanisms for regulating body temperature through behavioral thermoregulation have been described. The relevant genes have been elucidated through behavioral genetic analyses of *Drosophila* temperature preferences using temperature gradient or two-temperature choice assays (Suito et al., 2022). Typically, *Drosophila* avoid temperatures below 18°C and above 25°C, but flies having mutations in temperature receptor genes avoid neither high nor low temperatures. Thus, the activities of temperature receptors tightly link to the behavioral thermoregulation in *Drosophila*.

TRPA1 is the most-analyzed temperature receptor in insects, and both TRPA1 and TRPA subfamily proteins function as heat sensors not only in fruit flies but also in mosquitoes, bees, and silk moths (Matsuura et al., 2009). Five TRPA1 splicing variants (TRPA1-A to E) have been identified in fruit flies (Gu et al., 2019). Among these variants, TRPA1-A and -D show clear temperature sensitivity and functionality in sensory neurons. TRPA1-A has an activation temperature threshold of around 25°C (Hamada et al., 2008) and is expressed in the central nervous system where it contributes to the temperature preference of larvae and adults (Rosenzweig et al., 2005; Hamada et al., 2008). Warmth-induced rolling behavior of *Drosophila* is also regulated by TRPA1-A and TRPA1-B (Luo et al., 2017) as well as temperature preference of larvae. TRPA1-C and TRPA1-D are expressed in peripheral multi-dendritic sensory neurons that respond to nociceptive stimuli (Zhong et al., 2012; Gu et al., 2019). TRPA1-C and TRPA1-D activation temperature thresholds are higher than that of TRPA1-A (Zhong et al., 2012). Roles for members of the TRPA subfamily, Painless (Pain) (Tracey et al., 2003) and Pyrexia (Pyx) (Lee Y. et al., 2005), have been identified in the detection of noxious heat. In larvae, TRPL (Rosenzweig et al., 2008), which belongs to the TRPC subfamily, and Inactive (Iav) (Kwon et al., 2010) of the TRPV subfamily, are involved in cold perception, whereas in adults, TRPP subfamily member Brivido (Brv) (Gallio et al., 2011) has a role. NompC (TRPN subfamily), TRPM, and Amo (TRPP subfamily) are implicated in noxious cold perception (Turner et al., 2016). Additionally, roles in temperature reception have been demonstrated for Gr28b(D) (Ni et al., 2013) of the gustatory receptor family and ionotropic receptors such as Ir21a, Ir25a, and Ir93a (Tyrrell et al., 2021) and Ir21a, Ir25a, and Ir68a (Hernandez-Nunez et al., 2021). The activation of these thermoreceptor-expressing neurons causes the avoidance of unfavorable temperatures either extreme hot or cold, which generate the changes of temperature preference in *Drosophila*.

Comparative biological analyses of mosquitoes, which are in the same order (*Diptera*) as *Drosophila*, revealed that differences in TRPA1 activation temperature thresholds correlate with adaptation to environments having different temperatures. CpTRPA1, the temperature receptor of *Culex pipiens pallens* that inhabits regions with moderate temperatures like those in Japan, is activated above 21°C, such that these mosquitoes avoid 30°C for the two-way choice temperature preference assay. On the other hand, AaTRPA1, the temperature receptor of *Aedes aegypti* that inhabits tropical regions, is activated above 32°C; these mosquitoes do not avoid 30°C for the preference assay (Li et al., 2019). Further functional analysis of chimeric proteins of AaTRPA1 and CpTRPA1 revealed that differences in activation temperature thresholds are related to specific amino acid residues in the N-terminal region (Nguyen et al., 2022). This evidence indicates that the temperature sensitivity of the thermo-TRPs evolutionally couples with the adaptation to thermal niches.

The activity of TRPA1-expressing neurons and thermoregulation behavior in *Drosophila* are reported to be modulated by various signaling processes. TRP (a member of the TRPC subfamily that was the first trp protein identified in *Drosophila*) expressed in *Drosophila* visual cells is activated downstream of the photoreceptor rhodopsin, a G protein-coupled receptor, and phospholipase C (PLC) signaling cascades (Hardie, 2011). PLC signals are also reported to be necessary for activation of TRPA1-dependent thermoregulatory behavior (Kwon et al., 2008). Intriguingly, an involvement of rhodopsins in thermosensation has also been revealed. Several rhodopsins (e.g., Rh5, Rh6) are expressed in TRPA1-expressing neurons (Sokabe et al., 2016). Moreover, these rhodopsins are involved in developmental stage-dependent changes in the preferred temperature of larvae that falls within a range between 18°C and 25°C (Sokabe et al., 2016). In adults, the neuronal activity in TRPA1-expressing neurons is directly regulated by insulin-like peptide signaling, which is related to a decrease in the preferred temperature that is induced by nutritional starvation (Umezaki et al., 2018). Thus, various internal signals tune behavioral thermoregulation via the direct modulation of the sensory neuron activity.

## 5 Membrane lipids as modulators of thermosensitive TRP channels

TRP channels, including the thermosensitive TRP channels, are known to interact with membrane lipids like phosphoinositide (Taberner et al., 2015). Lipid mediators derived from cell membrane lipids also directly activate TRP channels as was shown for mammalian TRPV activation by endocannabinoids such as 2-AG (2-arachidonoyl glycerol) and N-acylethanolamines (Sisignano et al., 2014). In *Drosophila*, a recent study showed that 2-LG (2-linoleoyl glycerol), endocannabinoid derived from linoleic acid-containing phospholipids, activate TRP and TRPL, which is involved in visual signaling in the compound eye (Sokabe et al., 2022).

On the other hand, cell membrane properties are also reported to regulate the function of TRP channels (Cordero-Morales and Vasquez, 2018; Stieger et al., 2021). The composition of cell membranes determines its properties. For example, polyunsaturated fatty acids, which have multiple kinks within the molecular structure, contribute to maintaining membrane fluidity as shown for cell membrane phospholipids carrying polyunsaturated fatty acids. Indeed, living organisms inhabiting low temperatures show increased amounts of polyunsaturated fatty acids in cell membrane lipids that presumably allow better adaptation to cold environments. In *Drosophila*, TRPA1-activity is reported to be modulated by phospholipids-containing polyunsaturated fatty acids (Suito et al., 2020). Overexpression of Δ12 fatty acid desaturase enzymes that are involved in synthesizing linoleic acid from oleic acid has been shown to alter temperature preference and, TRPA1-expressing neurons in *Drosophila*. Pan-neuronal ectopic expression of the Δ12 fatty acid desaturase gene FAT-2 in *Caenorhabditis elegans* using a GAL4-UAS system induced a decrease in the preferred temperature in third instar larvae. Furthermore, ectopic expression of FAT-2 solely in TRPA1-expressing neurons also caused a decrease in preferred temperature. Analysis of TRPA1-expressing neurons in the brain using *ex vivo* calcium imaging showed that FAT-2 expression increased the maximum activation of TRPA1-expressing neurons to warm temperature stimuli, suggesting that modulation of thermosensory neuron activity induces a decrease in the preferred temperature in *Drosophila*. Although the absence of desaturase enzymes that produce polyunsaturated fatty acids, *Drosophila* have the Δ9 fatty acid desaturase DESAT1, which is involved in synthesizing monounsaturated fatty acids. DESAT1 is important for maintaining mitochondrial function and contributes to autonomous regulation of intracellular temperature (Murakami et al., 2022). Suppression of DESAT1 expression in the fat body, tissue that plays a central role in lipid synthesis and storage in *Drosophila*, led to an increase in the preferred temperature (Suito et al., 2022), indicating that changes in intracellular temperature and energy metabolism in mitochondria by modulation of monounsaturated fatty acids may not only modify membrane properties, but also affect temperature preference.

Recently, an effect of ether phospholipids on TRPA1 channel activity was demonstrated. *Drosophila* lacking the gene encoding AGPS, an enzyme involved in ether lipid synthesis, exhibit abnormalities in sensation of hot temperatures that results in an increase in preferred temperature (Suito et al., 2023). Suppressing AGPS expression in TRPA1-expressing neurons also produces similar phenotypic patterns. Electrophysiological analysis using TRPA1 expressed in *Drosophila* cell lines showed that ether lipids can alter the activation temperature threshold of TRPA1 channels. Ether lipids alter membrane tension and lipid order phase of cell membranes (Suito et al., 2023). Thus, the physicochemical properties of cellular membranes may determine sensory properties.

## 6 Discussion

This review focuses on the function of thermosensitive TRPs and considers factors that control behavioral thermoregulation in mice and *Drosophila*. Mice with thermo-TRP channel knockout display abnormalities in thermoregulatory behaviors that are mediated by peripheral thermosensitive neurons. However, in actual behavioral thermoregulation of homeothermic animals, various factors such as the relationship between physiological thermoregulation and emotion linked to thermosensation must be considered in order to better understand the thermoregulatory system. Furthermore, the degree to which these temperature receptors contribute to maintaining body temperature remains unclear. Indeed, the lack of obvious change in body temperature seen for mice with knockout of thermosensitive TRP may be due in part to the action of intrinsic temperature receptor molecules in the central nervous system such as hypothalamic thermosensitive neurons that are currently unknown. TRPM2 in the anterior hypothalamus was recently shown to have a role in controlling thermoregulation during fever (Song et al., 2016). TRPC4 was shown to be involved in regulating intrinsic body temperature in the preoptic area of the hypothalamus, even though TRPC4 has not been shown to exhibit thermosensitivity (Zhou et al., 2023). In the future, identifying the temperature sensor molecules involved in body temperature determination can provide a better understanding of the molecular mechanisms that govern thermoregulation in homeothermic animals.

**Figure 2:**
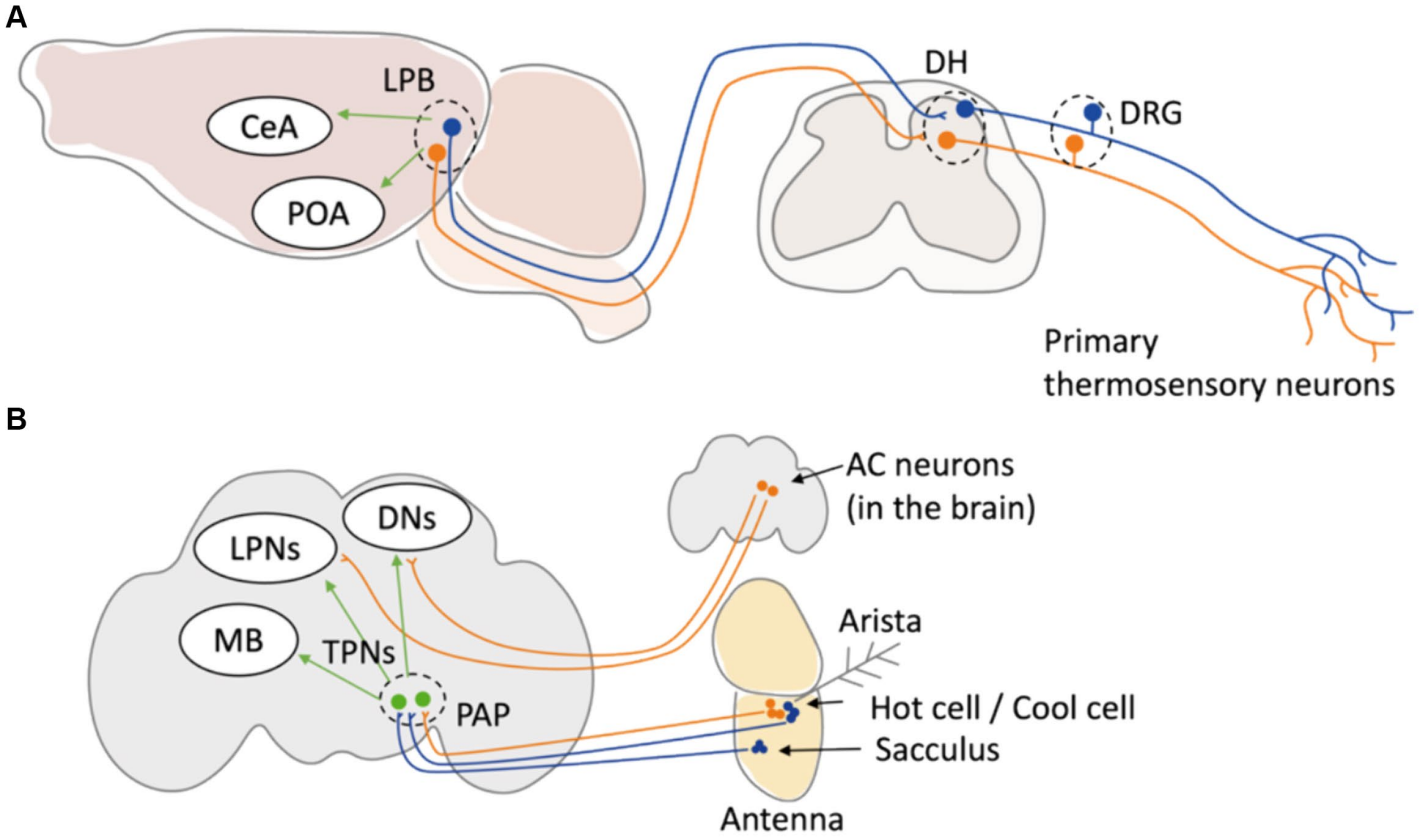
Neuronal circuit of thermal sensation in mice and *Drosophila*. **(A)** In mice, primary thermoreceptor neurons in DRG relay temperature information to LBP in the brain stem through DH of spinal cord. The temperature information is then sent to POA to induce physiological thermoregulation. The neuron projecting from LPB to MnPO and to CeA have been elucidated as neural pathways involved in behavioral thermoregulation. **(B)** In adult Drosophila, primary thermosensory neurons in the arista (Hot cells and cool cells) and in the sacculus project to the TPNs at PAP in the brain. TPNs relay the information to central part of the brain such as DNs, LPNs, and MB. In addition, TRPA1-expressing anterior cell (AC) neurons located in the brain central transfer temperature information to DNs and LPNs. DRG: dorsal root ganglion. DH: dorsal horn. LPB: lateral parabrachial nucleus. POA: preoptic area. CeA: central amygdala. PAP: proximal antennal protocerebrum. MB: mushroom body. DNs: dorsal neurons. LPNs: lateral posterior neurons.

Although the involvement of peripheral thermosensation in behavioral thermoregulation has been elucidated, the mechanism by which sensory information is integrated within the brain during behavioral thermoregulation is still unclear. Recently, the neural circuit projecting from the lateral parabrachial nucleus (LPB) to the median preoptic nucleus (MnPO) in response to heat and the pathway projecting to the central amygdala (CeA) in response to cold have been elucidated as neural pathways involved in both behavioral and physiological thermoregulation (Yahiro et al., 2023) (Figure 2A). In *Drosophila*, the circuit mechanisms of thermosensory processes in the brain are better understood compared to those in mammals (Mizunami et al., 2016). Signals of warm and cold temperatures from primary sensory neurons in *Drosophila* antenna are projected to distinct secondary neurons so called thermosensory projection neurons (TPNs) and finally processed in central brain regions like the mushroom body (MB) or dorsal neurons (DNs) (Frank et al., 2015; Liu et al., 2015; Alpert et al., 2020; Marin et al., 2020; Alpert et al., 2022) (Figure 2B), which mediates various physiological functions including learning and sleep. This independent integration of warm and cold temperature signals has also been observed in mammals (Vestergaard et al., 2023). Recent studies using connectomics approaches are beginning to unveil how external temperature information is integrated within the brain, and such integration may be conserved in many other organisms. Further elucidation of the mechanisms associated with behavioral thermoregulation is expected to progress through analyses using model organisms.

## Author contributions

TS: Writing – original draft, Writing – review & editing. MT: Writing – original draft, Writing – review & editing.
